# Identifying Diagnostic and Prognostic Biomarkers and Candidate Therapeutic Drugs of Gastric Cancer Based on Transcriptomics and Single-Cell Sequencing

**DOI:** 10.3389/pore.2021.1609955

**Published:** 2021-11-25

**Authors:** Xu Zhao, Shuang Wu, Jingjing Jing

**Affiliations:** ^1^ Mathematical Computer Teaching and Research Office, Liaoning Vocational College of Medicine, Shenyang, China; ^2^ College of Computer Science and Technology, Changchun Normal University, Changchun, China; ^3^ Tumor Etiology and Screening Department of Cancer Institute and General Surgery, The First Hospital of China Medical University, Shenyang, China; ^4^ Key Laboratory of Cancer Etiology and Prevention in Liaoning Education Department, The First Hospital of China Medical University, Shenyang, China; ^5^ Key Laboratory of GI Cancer Etiology and Prevention in Liaoning Province, The First Hospital of China Medical University, Shenyang, China

**Keywords:** bioinformatics, biomarkers, prognosis, gastric cancer, hub genes, molecular drugs

## Abstract

**Background and Objective:** Gastric cancer (GC) is an important health burden and the prognosis of GC is poor. We aimed to explore new diagnostic and prognostic indicators as well as potential therapeutic targets for GC in the current study.

**Methods:** We screened the overlapped differentially expressed genes (DEGs) from GSE54129 and TCGA STAD datasets. Protein-protein interaction network analysis recognized the hub genes among the DEGs. The roles of these genes in diagnosis, prognosis, and their relationship with immune infiltrates and drug sensitivity of GC were analyzed using R studio. Finally, the clinically significant hub genes were verified using single-cell RNA sequencing (scRNA-seq) data.

**Results:** A total of 222 overlapping genes were screened, which were enriched in extracellular matrix-related pathways. Further, 17 hub genes were identified, and our findings demonstrated that BGN, COMP, COL5A2, and SPARC might be important diagnostic and prognostic indicators of GC, which were also correlated with immune cell infiltration, tumor mutation burden (TMB), microsatellite instability (MSI), and sensitivity of therapeutic drugs. The scRNA-seq results further confirmed that all four hub genes were highly expressed in GC.

**Conclusion:** Based on transcriptomics and single-cell sequencing, we identified four diagnostic and prognostic biomarkers of GC, including BGN, COMP, COL5A2, and SPARC, which can help predict drug sensitivity for GC as well.

## Introduction

Gastric cancer (GC) is an important health burden and the third leading cause of cancer death worldwide [1]. Although radical surgery combined with perioperative management of GC has improved, the survival rates of most advanced GC patients are still very low [2]. Despite the recognition of the molecular mechanisms of GC and the significant progress in the implementation of new treatment strategies including immune and targeted therapy, not all patients respond to the existing therapy methods based on the recognized biomarkers [3, 4]. Therefore, it is of great significance to identify novel risk and prognostic markers in order to improve the early detection and effective treatment of GC.

Recently, the technology development of microarray and high-throughput sequencing has provided an effective tool for the identification of key genes in the process of tumor development and prognosis [5]. At the same time, in order to overcome the limitations or inconsistencies of data from different platforms or small sample research, integrated bioinformatics analyses can help to find much more valuable bioinformation [6, 7]. In the current study, we first combined data from a microarray and RNA sequencing array to analyze and identify the differential expression genes (DEGs) between human GC and noncancerous gastric tissues. Further, functional and pathway enrichment analyses were carried out to investigate the biological function regulation of DEGs. We constructed protein–protein interaction (PPI) networks, and the hub genes with high degrees of connectivity were identified. Receiver operating characteristic curve (ROC) as well as survival analysis were conducted to evaluate the diagnostic and prognostic values of the hub genes. Association of immune cell infiltration and drug sensitivity with the hub genes were further evaluated. Finally, the clinically significant hub genes were verified using single-cell RNA sequencing (scRNA-seq) data. This study may help to advance the understanding of diagnosis, prognosis, and treatment of GC.

## Materials and Methods

### Expression Data

We obtained the gene expression profiling microarray (GSE54129) from the Gene Expression Omnibus (GEO; https://www.ncbi.nlm.nih.gov/geo/) database. A total of 111 primary GC samples and 21 noncancerous gastric tissues were measured in this array (Platform: GPL570 Affymetrix Human Genome U133 Plus 2.0 Array). By consulting the Xena Functional Genomics Explorer of the University of California Santa Cruz (https://xenabrowser.net/) [8] and the STAD dataset of The Cancer Genome Atlas (TCGA), the expression data information of 478 tumor samples and 102 noncancerous controls were downloaded. The information of the samples is shown in Supplementary File S1. GEPIA (http://gepia.cancer-pku.cn/) is a database that uses standard processing methods to analyze the RNA sequencing expression data of 9,736 tumors and 8,587 normal samples from the TCGA and GTEx projects [9]. Multiple-gene comparison was conducted using GEPIA.

For scRNA-seq analysis, a total of six samples from six patients were analyzed in this study, including three normal and three GC samples. Data were downloaded from two sets of raw scRNA-seq data. GSE134520, comprising three normal samples and one GC sample, was included. Another dataset with the database of Genotypes and Phenotypes (dbGaP) accession number, phs001818.v2, comprising two GC cases, was included. The clinicopathologic parameters of the patients are presented in the supplementary tables (Supplementary Table S1 for GSE134520, Supplementary Table S2 for phs001818.v2).

### Data Processing

Using GEO2R, an online software, we analyzed the raw data of the microarray in GSE54129 to identify the DEGs. The TCGA STAD dataset was processed by R studio version 1.1.463, using the TCGA-Biolinks package. The cut-off criteria were defined as *p* value < 0.05 and |FC| > 1.5. Further, the online tool jvenn (http://jvenn.toulouse.inra.fr/app/index.html) [10] was adopted to find the overlapping DEGs of the two datasets of gene expression. The increased and the decreased genes were measured separately.

The quality control (QC) process of scRNA-seq data was performed using Seurat (version 3.0.1). A raw unique molecular identifier (UMI) count matrix was produced and converted into a Seurat object. Our results showed that sequencing counts were negatively correlated with mitochondrial percentage levels and positively related to sequencing features. UMI counts from single cells whose UMI number was <400, and the percentage of mitochondrial-derived UMI counts >20 were deleted. To optimally eliminate potential doublets, single cells containing >7,000 genes were also filtered out. Then, using the “NormalizeData” function, single-cell gene expression data were normalized, and the normalization method was set to “LogNormalize”. Finally, we used the corrected expression matrix as an input for further studies.

### Gene Ontology, Kyoto Encyclopedia of Genes and Genomes, and Gene Set Enrichment Analysis

Gene Ontology (GO) is a common bioinformatics tool, which is widely used to unify and annotate the representation of genes and proteins [11]. The description of cellular function is based on three major categories: cellular component, molecular function, and biological process. KEGG (Kyoto Encyclopedia of Genes and Genomes) is a database integrating genes and genomes and information about genomes, biological pathways, diseases, and chemicals [12]. We conducted GO and KEGG pathway enrichment analyses using R package Cluster profiler. Gene set enrichment analysis (GSEA) was performed using WebGestalt (WEB-based Gene SeT AnaLysis Toolkit, http://www.webgestalt.org/) [13]. *p* < 0.05 was considered statistically significance.

### Protein–Protein Interaction Network and Co-Expression Analysis

The functional interaction between proteins is very important for understanding the metabolism and molecular mechanism of tumord. Search Tool for the Retrieval of Interacting Genes (STRING) (https://string-db.org/) can help collect and integrate the known and predicted protein-protein association data [14]. Using STRING, the protein–protein interactions (PPI) network was constructed and visualized according to the overlapped DEGs-coded proteins. The threshold was defined as interaction score = 0.4. Subsequently, module clustering analysis was conducted by Molecular Complex Detection (MCODE) and cytoHubba in the Cytoscape software [15]. MCODE score >6 and number of nodes ≥3 were selected as the screening criteria. Genes were defined as hub genes when the connection degree >10. A multi-gene correlation map was generated by the R software package heatmap. We used Spearman’s correlation analysis to describe the correlation between hub genes. A *p*-value of less than 0.05 was considered statistically significant.

### Survival and Clinical Data

The TCGA clinical and survival information was obtained from Xena Functional Genomics Explorer of University of California Santa Cruz (https://xenabrowser.net/) [16], and along with the expression data, they were analyzed by R studio. According to the quartile value of gene expression, patients were divided into a low expression group and high expression group. If the gene expression was greater than or equal to the lower quartile, it was defined as high expression, otherwise it was defined as low expression.

### Construction of the Prognostic Model Based on the Hub Genes

The R package “glmmet” was used for model fitting; four identified genes were used as independent variables to form the model, and the corresponding parameters of each gene were calculated. We used ridge regression to retrieve the coefficient of each gene, and then all gene coefficients were calculated by multiplying their gene expression to get a new risk factor. Univariate and multivariate Cox regression analyses were carried out with other common clinical risk factors. Finally, the independent risk factors affecting GC were obtained, and the nomogram was constructed using these risk factors.

### Correlation Analysis of Hub Gene Expression and Immune Infiltration

TIMER (https://cistrome.shinyapps.io/timer/) is a comprehensive resource for systematic analysis of tumor-infiltrating immune cells across 32 different cancers from the TCGA database [17]. Using TIMER, we evaluated the associations between hub genes expression and immune cell populations (B cells, CD8+ T cells, CD4+ T cells, macrophages, neutrophils, and dendritic cells) in GC.

### Correlation Analysis of Hub Gene Expression and Tumor Mutation Burden/Microsatellite Instability

TMB and MSI are important predictive markers of immunotherapy. TMB and MSI data were from the TCGA database. TMB is defined as the total mutation rate per million base pairs. MSI is defined by counting the number of insertion or deletion events that occur in the repeated sequences of genes. In order to explore the correlation between hub genes and TMB/MSI, we calculated the Spearman’s correlation coefficient between the expression of hub genes and TMB/MSI score using R studio. A *p*-value of less than 0.05 was considered statistically significant.

### Drug Sensitivity Analysis

We downloaded the NCI-60 drug sensitivity Z scores and corresponding NCI-60 cell lines RNA-seq expression data from the CellMiner database (https://discover.nci.nih.gov/cellminer/home.do). The higher the cell line Z score, the more sensitive it is to the corresponding drugs. For better clinical applications, only FDA-approved drugs and drugs under clinical trials were included in the analysis. Spearman’s correlation analysis was performed to determine the correlation using R studio.

### Statistical Analysis

The association between the gene expressions and clinical features was evaluated by Pearson’s X^2^ test. The Kaplan-Meier method was used to evaluate the correlation between gene expression and total survival time, and the log rank test was used for comparison. Univariate and multivariate Cox proportional hazards models were used to estimate the effect on overall survival with or without adjustment for confounding factors. A multivariate Cox proportional risk regression model further adjusted age, gender, grade, and TNM stage to evaluate the independent prognostic value. A Sankey diagram was built based on the R software package ggalluval. All statistical analyses were performed by R studio. There was a significant difference between the two groups when *p* < 0.05.

## Results

### DEGs Between GC and Normal Tissues Based on the GEO and TCGA Database

**FIGURE 1:**
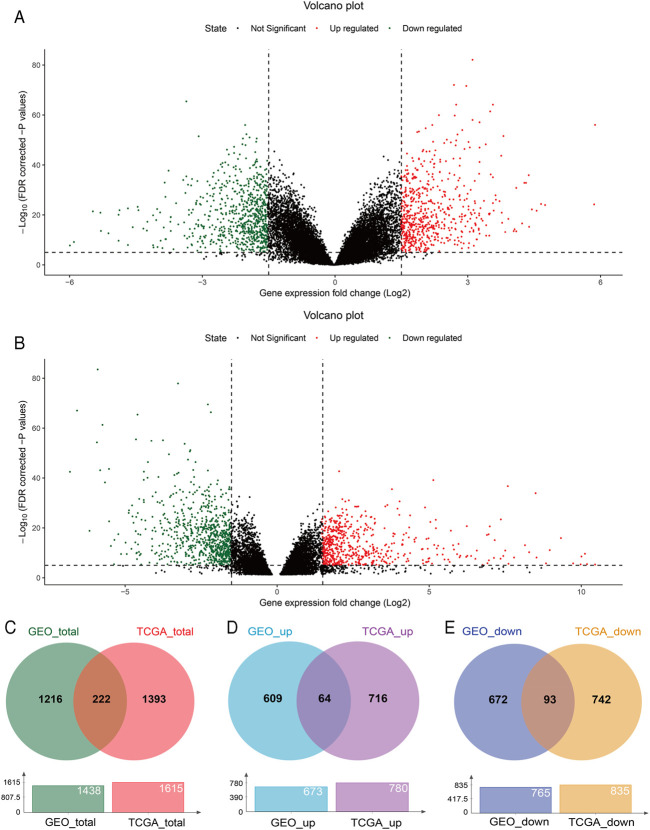
DEGs between GC and normal tissues based on the GEO and TCGA database. Volcano plot of DEGs screened from the GSE54129 microarray dataset **(A)** and TCGA STAD **(B)**. Red: upregulated; green: downregulated. Overlapping DEGs from the GEO and TCGA database **(C)**; upregulated and downregulated overlapping genes from the GEO and TCGA database **(D,E)**.

With GEO2R, 673 upregulated and 765 downregulated DEGs were screened from the GSE54129 microarray dataset for further analysis (Figure 1A). The TCGA STAD dataset was analyzed using the TCGA-Biolinks package of R studio, 780 upregulated and 835 downregulated genes were identified (Figure 1B). Totally, 1438 and 1615 DEGs were separately identified from the GEO and TCGA database, with 222 overlapping DEGs (Figure 1C), of which 64 upregulated and 93 downregulated overlapping genes were also identified (Figures 1D,E). In addition, 8 genes were upregulated in TCGA but downregulated in the GEO, while 57 genes were upregulated in the GEO but downregulated in TCGA (Supplementary Figure S1).

### GO, KEGG, and GSEA Enrichment Results

**FIGURE 2:**
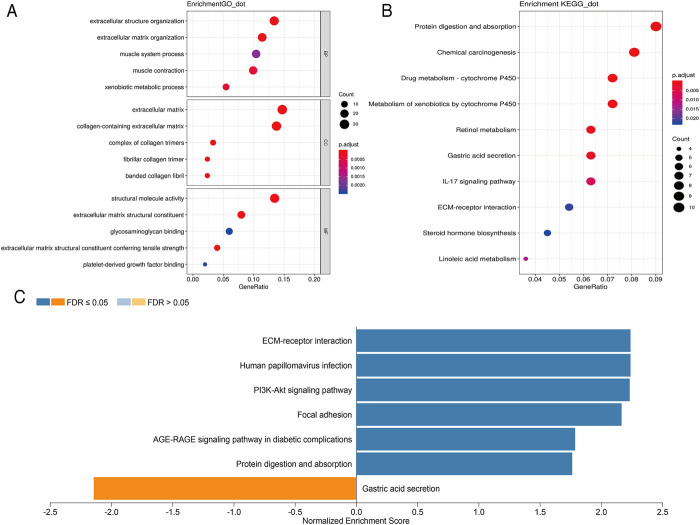
Scatterplots for GO enrichment and KEGG pathways of DEGs. **(A)** GO enrichment of DEGs; **(B)** KEGG pathways of DEGs. The gene ratio is assigned to the *x*-axis and the description of pathway to the *y*-axis. The area of the displayed circles is proportional to the number of genes assigned to the term and the color corresponds to the adjusted *p*-value. **(C)** GSEA analysis of DEGs. The bar chart displays the normalized enrichment score of pathways that were significantly related to DEGs.

In order to further analyze the biological function of DEGs, we carried out GO and KEGG pathway enrichment analyses. We first conducted GO function analysis. Regarding biological process (BP), the DEGs were enriched in extracellular structure organization, extracellular matrix organization, muscle contraction, xenobiotic metabolic process, and muscle system process. As for cellular component (CC), collagen-containing extracellular matrix, extracellular matrix, complex of collagen trimers, fibrillar collagen trimer, and banded collagen fibril were identified. Concerning molecular functions (MF), enrichment was found in extracellular matrix structural constituent, extracellular matrix structural constituent conferring tensile strength, structural molecule activity, platelet-derived growth factor binding, and glycosaminoglycan binding (Figure 2A). These results were shown in Supplementary Table S3. KEGG pathway analysis showed that these DEGs were mainly enriched in protein digestion and absorption, chemical carcinogenesis, drug metabolism-cytochrome P450, metabolism of xenobiotics by cytochrome P450, and retinol metabolism (Figure 2B). The 10 pathways with *p* value < 0.05 are available in Supplementary Table S4. GSEA analysis showed that DEGs were closely related to extracellular matrix (ECM)-receptor interaction, human papillomavirus infection, PI3K-Akt signaling pathway, focal adhesion, AGE-RAGE signaling pathway in diabetic complications, protein digestion and absorption, and gastric acid secretion (Figure 2C).

### PPI Network and Co-Expression of Hub Genes

**FIGURE 3:**
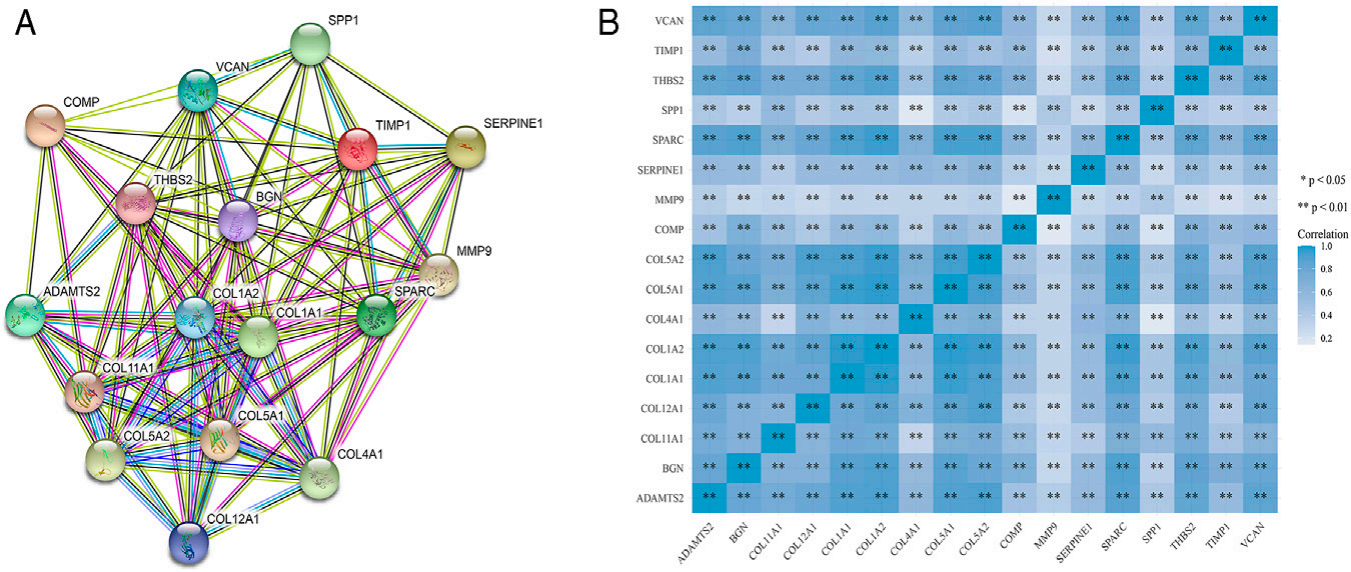
The PPI interaction network and co-expression based on the hub genes. **(A)** The PPI interaction network of the hub genes-coded proteins. Nodes represent proteins and edges represent interactions between two proteins. **(B)** The heatmap of the co-expression of the hub genes. The darker the color, the stronger the correlation. Asterisks represent levels of significance (*p < 0.05, **p < 0.01).

STRING was used to construct interaction networks among the DEGs-coded proteins, which was helpful to further explore the relationships between DEGs at the protein level. Based on the screened overlapped DEGs, we obtained the PPI network by importing these genes into STRING. In the network, 17 hub genes were identified by Cytoscape (Table 1). Notably, these hub genes were all upregulated in overlapping DEGs. Subsequently, the interaction network between the proteins encoded by the hub genes was also constructed. These proteins had high degrees of connectivity (Figure 3A). Seventeen hub genes were all significantly correlated with each other (Figure 3B).

**TABLE 1:** The list of hub genes.

Hub genes	Degree of connectivity	MCODE score
ADAMTS2	12	6.8
BGN	20	7.5
COL11A1	14	6.7
COL12A1	14	7.6
COL1A1	31	6.4
COL1A2	26	6.4
COL4A1	18	6.4
COL5A1	15	8.1
COL5A2	16	6.7
COMP	13	7.0
MMP9	37	8.3
SERPINE1	18	7.8
SPARC	14	7.7
SPP1	23	7.8
THBS2	22	7.5
TIMP1	25	8.3
VCAN	22	6.6

### Hub Genes Were Associated With Clinicopathological Parameters of GC Patients

The relationship between the expression of hub genes and clinicopathological parameters of GC is summarized in Supplementary Table S5. High expression of COL1A2, BGN, COL5A1, COMP, and ADAMTS2 was related with worse pathological grade (*p* = 0.004; 0.005; 0.017; 0.030; <0.001). High expression of TIMP1, COL5A2, SPARC, and COL11A1 suggested deeper depth of invasion (T stage) (*p* = 0.017, 0.023, 0.031, 0.012, respectively). In addition, increased expression of VCAN obviously predicted worse (*p* = 0.031) T stage and worse pathological grade (*p* < 0.031).

### Hub Genes Were Associated With Prognosis of GC Patients

**FIGURE 4:**
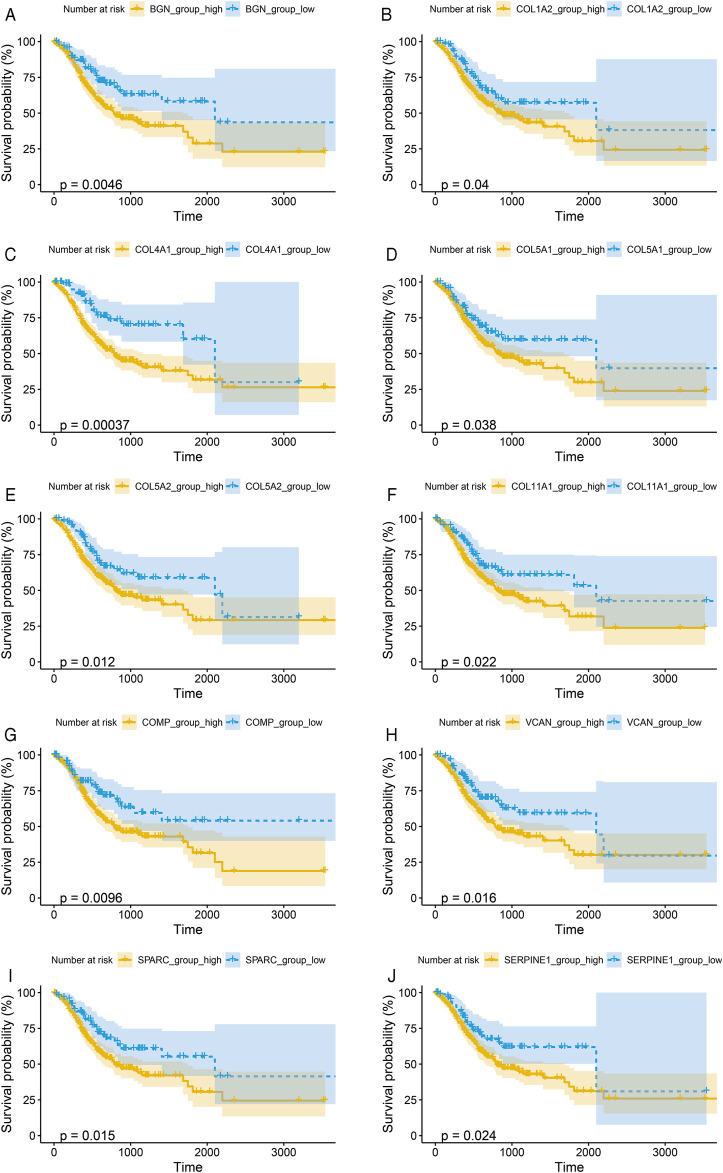
Kaplan-Meier survival curves by the expression level of hub genes. **(A)**, BGN; **(B)**, COL1A2; **(C)**, COL4A1; **(D)**, COL5A1; **(E)**, COL5A2; **(F)**, COL11A1; **(G)**, COMP; **(H)**, SERPINE1; **(I)**, SPARC; and **(J)**, VCAN. The patients were split into high and low expression groups according to the quartile value of the hub gene expression.

To verify the prognostic significance of these hub genes, we first performed Kaplan–Meier analysis using R studio. Our results showed that higher expression levels of BGN, COL1A2, COL4A1, COL5A1, COL5A2, COL11A1, COMP, SERPINE1, SPARC, and VCAN were associated with worse OS, whereas other genes were not significantly relevant (Figures 4A–J).

Further, we performed Cox proportional hazard ratio (HR) analysis to determine the prognostic value of the hub genes. Univariate analysis indicated that COL1A2, VCAN, BGN, SERPINE1, COL4A1, COL5A2, COL5A1, SPARC, COL11A1, and COMP expression levels were related to OS in GC patients (Table 2). To evaluate the independent prognostic value of the genes, univariate significant variables including age, TNM stage, and grade were further adjusted in multivariate analysis, respectively. Our results indicated that high expressions of BGN, COL4A1, COL5A2, SPARC, and COMP were associated with worse OS of GC (Table 2). The expression levels of these genes could be regarded as independent prognostic indicators of GC.

**TABLE 2:** Prognostic roles of hub genes’ mRNA expression in GC based on TCGA data.

Gene/variable	Univariate analysis		Multivariate analysis	
	HR (95% CI)	*p*	Adjusted HR (95% CI)	*p*
MMP9	0.901 (0.611–1.328)	0.597	0.904 (0.611–1.339)	0.616
COL1A1	1.368 (0.917–2.042)	0.125	1.290 (0.861–1.932)	0.217
COL1A2	**1.531 (1.016–2.306)**	**0.042**	1.441 (0.950–2.185)	0.086
TIMP1	1.368 (0.916–2.041)	0.126	1.355 (0.907–2.025)	0.137
SPP1	1.287 (0.859–1.929)	0.221	1.305 (0.870–1.958)	0.199
THBS2	1.409 (0.935–2.123)	0.101	1.312 (0.867–1.986)	0.199
VCAN	**1.655 (1.092–2.507)**	**0.017**	1.471 (0.960–2.255)	0.076
BGN	**1.824 (1.196–2.781)**	**0.005**	**1.742 (1.132–2.680)**	**0.016**
SERPINE1	**1.637 (1.061–2.524)**	**0.026**	1.449 (0.937–2.242)	0.096
COL4A1	**2.347 (1.446–3.809)**	**<0.001**	**2.207 (1.355–3.596)**	**0.001**
COL5A2	**1.678 (1.113–2.530)**	**0.013**	**1.543 (1.019–2.337)**	**0.040**
COL5A1	**1.547 (1.021–2.343)**	**0.039**	1.450 (0.954–2.202)	0.082
SPARC	**1.670 (1.102–2.530)**	**0.016**	**1.620(1.065–2.461)**	**0.024**
COL12A1	1.253 (0.836–1.878)	0.274	1.225 (0.816–1.838)	0.327
COL11A1	**1.619 (1.068–2.455)**	**0.023**	1.396 (0.912–2.137)	0.125
COMP	**1.747 (1.139–2.679)**	**0.011**	**1.672 (1.088–2.569)**	**0.019**
ADAMTS2	1.406 (0.934–2.119)	0.103	1.221 (0.798–1.869)	0.357
Age	**1.021 (1.006–1.037)**	**0.007**	**1.031 (1.014–1.048)**	**<0.001**
Gender	0.835 (0.596–1.171)	0.296	0.863 (0.607–1.228)	0.413
TNM	**2.066 (1.462–2.919)**	**<0.001**	**2.096 (1.470–2.988)**	**<0.001**
Grade	**1.426 (1.045–1.947)**	**0.025**	**1.432 (1.030–1.992)**	**0.033**

HR: hazard radio. Bold value indicates a significant prognostic role of the gene in gastric cancer.

### Four Clinically Significant Hub Genes Play a Diagnostic Role in GC

**FIGURE 5:**
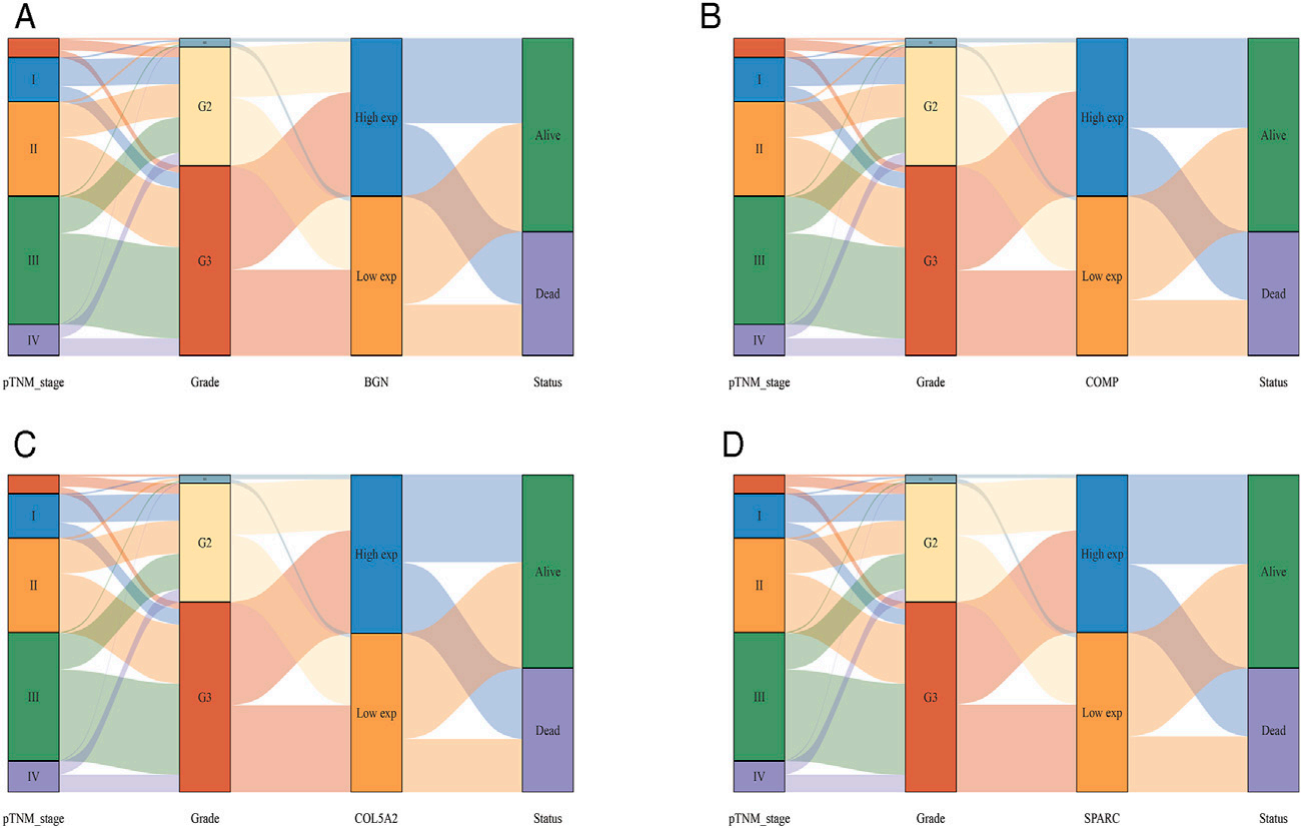
The Sankey diagram based on four hub genes with clinical and prognostic significance. **(A)**, BGN; **(B)**, COMP; **(C)**, COL5A2; and **(D)**, SPARC. Each column represents a characteristic variable, different colors represent different types, status, or stages, and lines represent the distribution of the same sample in different characteristic variables.

**FIGURE 6:**
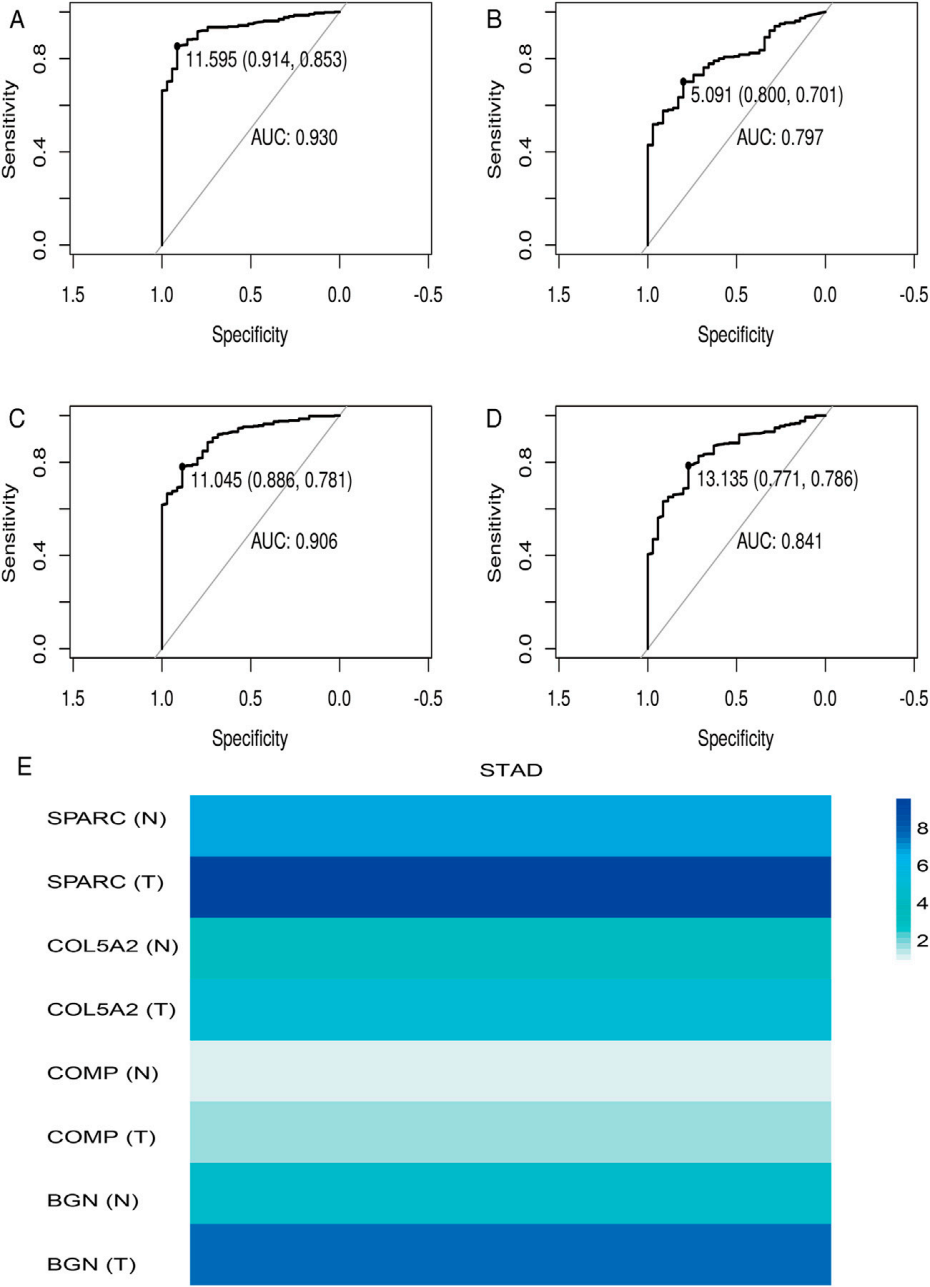
Diagnostic value of four hub genes in GC. **(A)**, BGN; **(B)**, COMP; **(C)**, COL5A2; **(D)**, SPARC; **(E)**, multiple-gene comparison analysis using GEPIA. AUC: area under curve; T: tumor; N: normal. The density of color in each block and the number on the right represent the median expression value of a gene in a given tissue, normalized by the maximum median expression value across all blocks.

According to the differential analysis and prognostic analysis, four genes were found to be highly expressed in GC and played a prognostic role as well, which may be clinically significant genes in GC. Therefore, we further focused on these four genes to explore their values. We visualized the clinical and prognostic significance of these four hub genes (BGN, COMP, COL5A2, and SPARC) in GC using a Sankey diagram (Figure 5). Most GC patients with high expression of the hub genes had advanced TNM stage, advanced pathological grade, and worse survival. Further, ROC curve analysis was used to determine the diagnostic value of these hub genes in GC. Our results showed that BGN (AUC = 0.930, *p* < 0.0001), COMP (AUC = 0.797, *p* < 0.0001), COL5A2 (AUC = 0.906, *p* < 0.0001), and SPARC (AUC = 0.841, *p* < 0.0001) can distinguish GC tissues from normal paracancerous mucosa (Figures 6A–D). Dramatically, all these genes have the potential for diagnosing GC. In addition, multiple-gene comparison analysis was conducted using GEPIA, it was further verified that the expression of these four genes in GC samples was also higher than that in normal samples (Figure 6E), indicating the potential of these genes in the diagnosis of GC.

### The Prognostic Model Based on the Four Hub Genes

**FIGURE 7:**
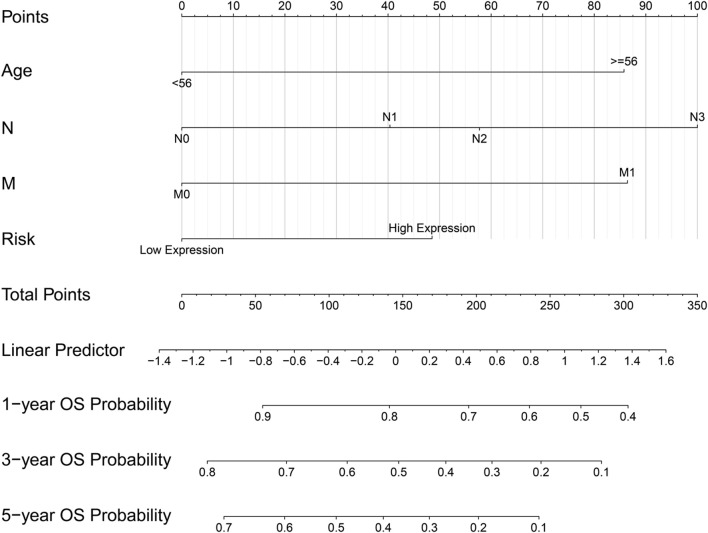
Nomogram based on four hub genes for predicting the probability of 1-, 3-, 5-years OS for GC patients of the TCGA cohort.

The ridge regression coefficients (BGN = −0.04476041; COMP = −0.03469480; COL5A2 = −0.09465680; SPARC = 0.39870514) of the selected genes were used to develop a novel prognostic biomarker (“risk”) for predicting the individual risk of GC progression. The nomogram based on the four genes and clinical features was constructed for predicting the 1-, 3- and 5-years overall survival of the GC patients (Figure 7). Therefore, they may be used for GC patients’ clinical management.

### Four Hub Genes Were Associated With Immune Cell Infiltration and TMB/MSI

**FIGURE 8:**
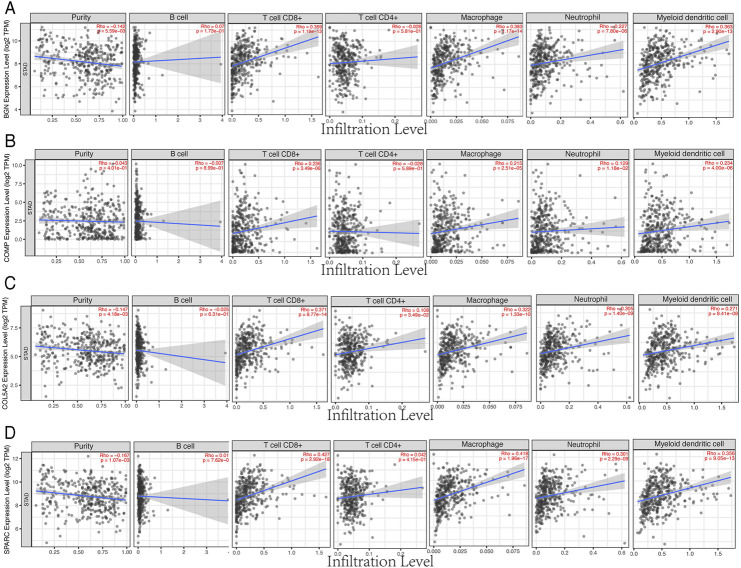
Correlations between four hub genes’ expression and immune infiltrates in GC. **(A)**, BGN; **(B)**, COMP; **(C)**, COL5A2; and **(D)**, SPARC.

**FIGURE 9:**
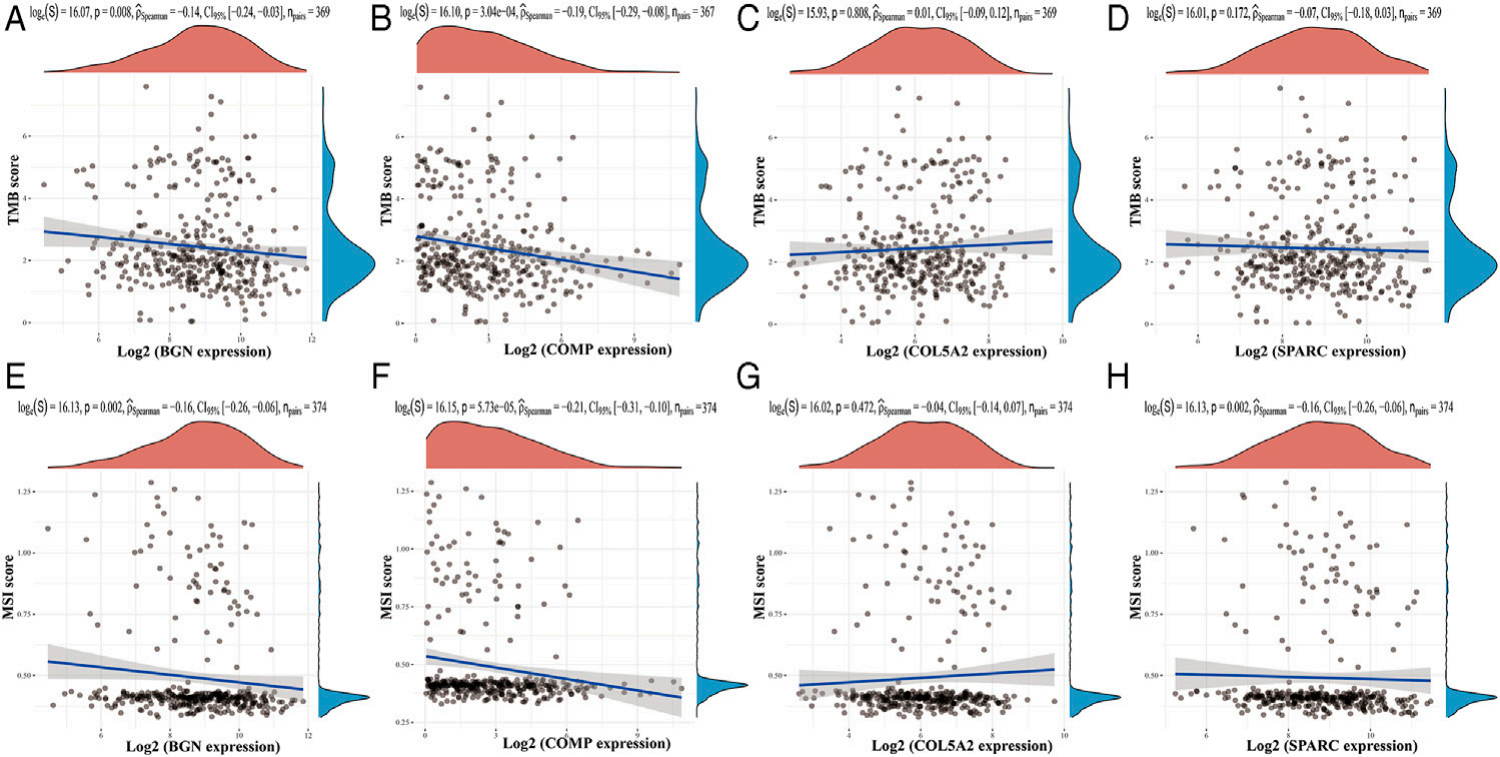
Correlations between four hub genes’ expression and TMB/MSI. **(A–D)**, Correlations between four hub genes’ expression and TMB; **(E–H)**, correlations between four hub genes’ expression and MSI. The horizontal axis in the figure represents the expression distribution of the gene, and the ordinate is the expression distribution of the TMB/MSI score.

Based on the TIMER database, the results showed that BGN, COMP, and SPARC were correlated with four types of immune cell infiltrates (CD8+ T cells, macrophages, neutrophils, and dendritic cells) to various degrees. COL5A2 was correlated with CD8+ T cells, CD4+ T cells, macrophages, neutrophils, and dendritic cells (Figure 8). Among the four hub genes, BGN and COMP were negatively correlated with TMB score, and BGN, COMP, and SPARC were negatively correlated with MSI score (Figure 9).

### Correlation Between the Drug Sensitivity and Expression of the Four Hub Genes

**FIGURE 10:**
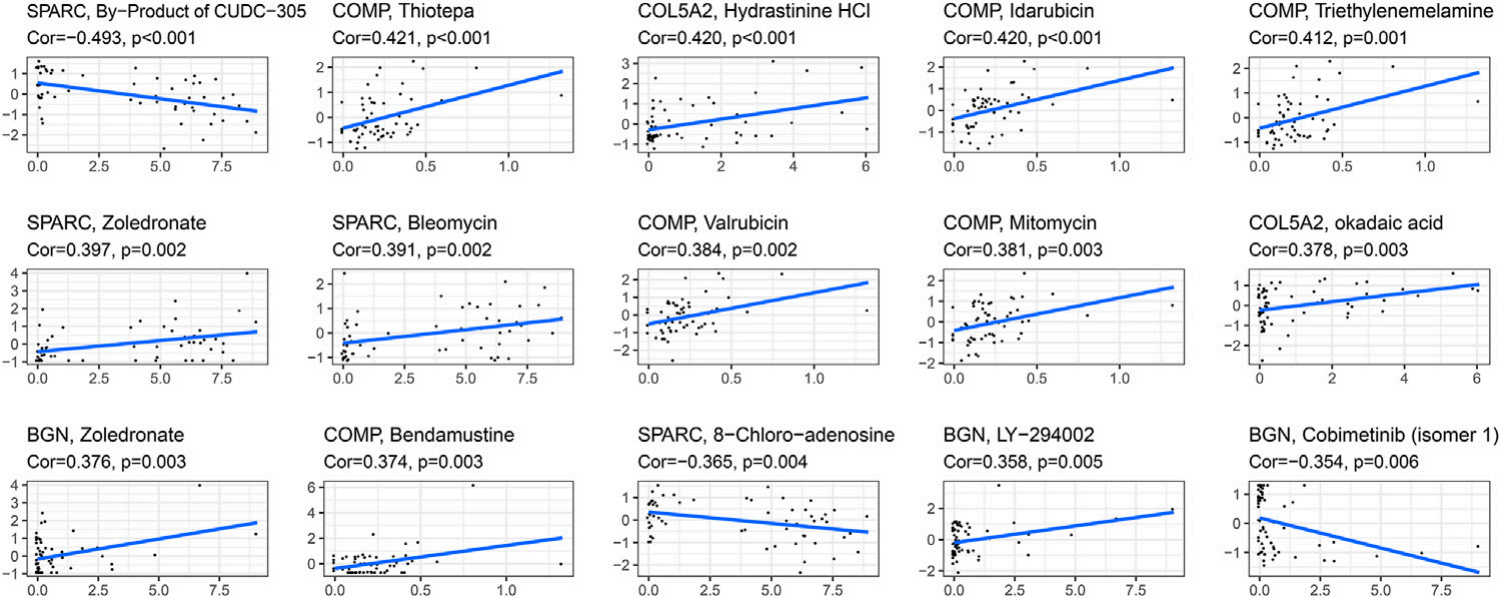
Correlations between four hub genes’ expression and drug sensitivity. The figure shows the top 15 significant drug-gene pairs with significant correlation. *X*-axis: gene expression; *y*-axis: drug sensitivity Z scores.

The correlation between the expression of the four hub genes and antitumor drug sensitivity was explored through the CellMiner database. Most drug sensitivity showed a positive correlation with gene expression. The most positively correlated drug of BGN, COMP, COL5A2, and SPARC was zoledronate, thiotepa, hydrastinine HCl, and zoledronate, separately. The by-product of CUDC-305, 8-chloro-adenosine, and cobimetinib showed highly significant negative correlation with gene expression. The correlation between the drugs and genes is summarized in Supplementary Table S6. The top 15 significant drug-gene pairs are shown in Figure 10.

### Verification of the Four Hub Genes’ Expression Using scRNA-seq Data

**FIGURE 11:**
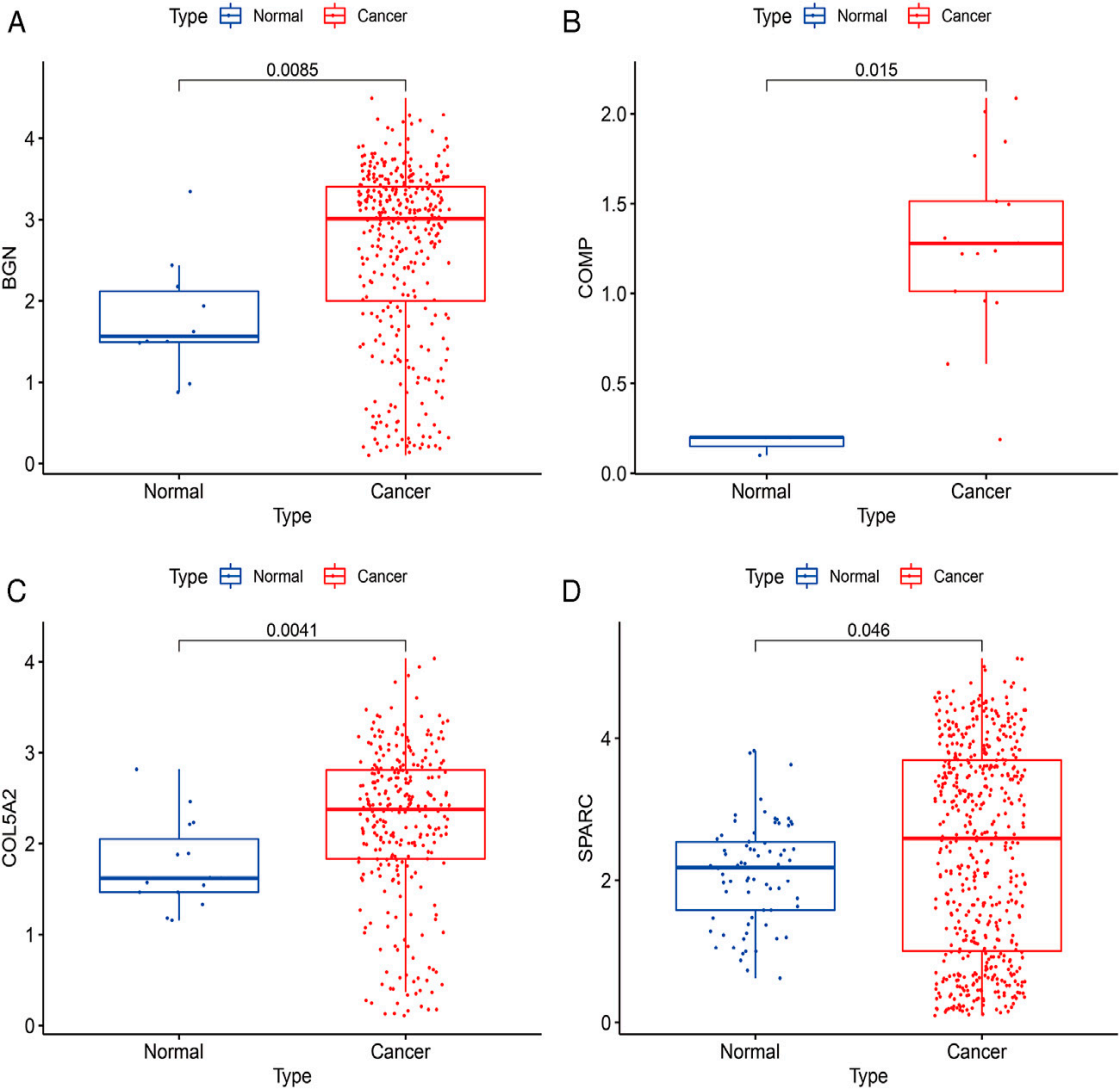
Verification results of four hub genes’ expression using scRNA-seq data. **(A)**, BGN; **(B)**, COMP; **(C)**, COL5A2; and **(D)**, SPARC.

The expressions of BGN, COMP, COL5A2, and SPARC were further verified using scRNA-seq data between cancer and normal samples. A total of 20370 single cells were obtained from normal and GC samples. After QC, 13839 cells remained (Supplementary Table S7). There were 377 BGN-expressing cells, of which only 10 were from normal tissues and 367 were from cancer tissues. There were 340 cells expressing COL5A2, of which only 15 cells were from normal tissues and 325 cells were from cancer tissues. There were 20 cells expressing COMP, of which only 3 cells were from normal tissues and 17 cells were from cancer tissues. There were 613 SPARC-expressing cells, of which only 73 were from normal tissues and 540 were from cancer tissues. These results further confirmed that all four hub genes were highly expressed in GC (Figure 11).

## Discussion

Although new diagnosis and treatment strategies have been implemented recently, it is still urgent and challenging to find new diagnostic markers, therapeutic targets, and methods. Previous studies have revealed a variety of biomarkers of GC, but their clinical values have not yet been fully confirmed. Our findings suggest that BGN, COMP, COL5A2, and SPARC are important clinical and prognostic indicators of GC. In addition, they can also be considered as diagnostic biomarkers of GC. These findings may provide new methods and targets for the diagnosis and treatment of GC, thus improving the prognosis of GC patients.

Firstly, a total of 222 overlapping DEGs between GC and normal tissues were screened from the GEO and TCGA database in the present research. The functional enrichment analysis demonstrated that these genes were mainly enriched in extracellular structure organization, extracellular matrix, structural molecule activity, etc. The results of KEGG enrichment analysis showed that the DEGs were mainly associated with protein digestion and absorption, chemical carcinogenesis, and drug metabolism-cytochrome P450, etc. GSEA analysis showed that DEGs were closely related to ECM-receptor interaction, human papillomavirus infection, PI3K-Akt signaling pathway, etc. Results of GO, KEGG, and GSEA analyses showed close relationships of the DEGs with ECM features. ECM is a network structure that is composed of collagen, glycoprotein, and proteoglycan. It is in a dynamic equilibrium under the influence of extracellular proteases and their inhibitors. It can regulate tissue development and cell homeostasis, and its imbalance is involved in cancer progression [18]. The cancer-associated ECM is not only an important feature of cancer but also plays an active role in cancer histopathology and behavior [19]. The above results indicated that those DEGs played an important role in ECM-related pathways. Interestingly, these DEGs are all ECM components or regulators. Therefore, the abnormal expression of these ECM-related genes and proteins may break the dynamic balance, which triggers pathological ECM remodeling, and results in reduced adhesion of cells to the ECM. This is conducive to cancer cells invading nearby organs and blood vessels, thereby promoting cancer progression, invasion, and metastasis. Therefore, our findings revealed the probable function and the regulation of these key genes in gastric carcinogenesis, which is worthy of further studies.

Based on these DEGs, our findings identified a set of biomarkers as potential diagnostic indicators of GC, which were also associated with clinical and prognostic characteristics in GC patients. Biglycan (BGN) is an important component of ECM proteins belonging to the small leucine-rich proteoglycans family, which has been reported to play an important role in the oncogenesis and progression of different cancers [20-22]. As for GC, Hu et al. found that BGN was secreted from GC cells into the tumor stroma, which may promote cancer progression through the chronic activation of tumor angiogenesis [23]. Another study from Wang et al. demonstrated that elevated expression of BGN could be evaluated as a biomarker for predication of a poor clinical outcome of GC [24]. Similarly, in our study, high expression of BGN was observed to be associated with worse clinical and prognostic parameters of GC, which suggested that BGN may take part in gastric carcinogenesis and behaviors.

Secreted protein acidic and rich in cysteine (SPARC) belongs to the family of matricellular proteins, which is necessary for calcification of the collagen in bone, synthesis of the extracellular matrix, and the promotion of changes to cell shape. It has been demonstrated that SPARC was overexpressed in some cancers, such as pancreatic carcinoma [25], esophageal squamous cell cancer [26], and GC [27]. In contrast, some other studies found that SPARC expression was reduced in bladder cancer [28] and acute leukemia [29]. It has been reported as a prognostic marker in many cancer types, such as breast cancer and melanoma [30]. In addition, Li et al. found that high expression of SPARC in GC was associated with a worse prognosis and might induce Adriamycin sensitivity in GC cells [31]. We also found that SPARC expression was increased in GC, and that a higher SPARC was related to deeper depth of invasion (T stage) and worse prognosis of GC. The results of other studies can partially confirm our results on the upregulation of SPARC in the development and progression of GC.

Thus far, the role of collagen type V alpha 2 chain (COL5A2) and cartilage oligomeric matrix protein (COMP) in GC has never been confirmed. In some bioinformatics studies, COL5A2 and COMP have been found to be associated with cancers including GC. For example, COL5A2 has previously been found to be associated with tumorigenesis, pathological processes or prognosis of osteosarcoma [32], bladder cancer [33], and GC [34]. Liang et al. [35] found that COMP is an upregulated methylation-regulated differentially expressed gene that is associated with clinical outcome of GC patients. Zhou et al. [36] observed that COMP was correlated with the recurrence of GC patients in stages III and IV accepting curative surgery plus chemoradiotherapy. Although the role of BGN, COMP, COL5A2, or SPARC in GC has been reported in other bioinformatics studies, in the current study, we focused on both the diagnostic and prognostic values of the four genes in GC. Interestingly, those four genes also have clinical value to serve as prognostic biomarkers of GC. Moreover, we further constructed a nomogram based on the four genes as a combining risk factor to predict GC prognosis. In addition, the four genes are all ECM components or regulators, revealing the probable function and the regulation of these key genes in gastric carcinogenesis, which is worthy of further study.

Further, we found that the expression of the four hub genes BGN, COMP, COL5A2, and SPARC was significantly correlated with immune cell infiltrates and purity, BGN and COMP were negatively correlated with TMB score, and BGN, COMP, and SPARC were negatively correlated with MSI score, which implied that these hub genes may play important roles in manipulating the GC immune microenvironment and immune therapy. To identify potential drugs for GC based on the four hub genes, we compared drug sensitivity of FDA-approved anticancer drugs. The positive correlation between drug sensitivity and the expression of the hub genes indicated that GC patients with a high expression of the hub genes were sensitive to the drug. The most positive correlated drugs were thiotepa, hydrastinine HCl, idarubicin, triethylenemelamine, etc., which can be conducive to GC treatment. The negative correlation may suggest that upregulation of the hub genes may affect the effect of the drug in GC. Therefore, the results suggested that the expression of these genes was helpful in predicting the sensitivity of cancer cells to these drugs. It also demonstrated that we can select drugs based on gene expression levels, which may provide a clue for more precise drug use. However, the correlation was established at the RNA level. Due to a lack of protein expression data, whether these genes would be potential therapeutic targets cannot be fully substantiated. Our results should be verified at the cellular and animal levels in future studies.

## Conclusion

In summary, based on transcriptomics and single-cell sequencing, our present study identified four potential biomarkers of GC, including BGN, COMP, COL5A2, and SPARC. These genes have the clinical value to serve as diagnostic and prognostic indicators, and to be used as a basis for drug sensitivity prediction for GC. Further investigation will be conducted to validate the function and mechanisms of these genes in the future.

## Data Availability

The original contributions presented in the study are included in the article/Supplementary Material, further inquiries can be directed to the corresponding authors.

## Abbreviations

GC, gastric cancer; DEGs, differentially expressed genes; PPI, protein–protein interaction; ROC, receiver operating characteristic curve; GEO, Gene Expression Omnibus; TCGA, The Cancer Genome Atlas; GO, Gene Ontology; KEGG, Kyoto Encyclopedia of Genes and Genomes; GSEA, gene set enrichment analysis; STRING, Search Tool for the Retrieval of Interacting Genes; MCODE, Molecular Complex Detection; BP, biological process; CC, cellular component; MF, molecular functions; HR, hazard ratio; ECM, extracellular matrix; BGN, biglycan; SPARC, secreted protein acidic and rich in cysteine; COL5A2, collagen type V alpha 2 chain; COMP, cartilage oligomeric matrix protein; QC, quality control.
