# Supplementary material for: Identifying Diagnostic and Prognostic Biomarkers and Candidate Therapeutic Drugs of Gastric Cancer Based on Transcriptomics and Single-Cell Sequencing
Source: Pathol Oncol Res. 2021 Nov 25;27:1609955. doi: 10.3389/pore.2021.1609955 (PMC8654733; doi:10.3389/pore.2021.1609955)
Supplement: Supplementary file 1 [file DataSheet1.docx]

| **TableS1. The clinicopathologic parameters of the patients in GSE134520** | | | | | | | | | | | | | |
| --- | --- | --- | --- | --- | --- | --- | --- | --- | --- | --- | --- | --- | --- |
| **Patient ID** | **Age** | **Sex** | **Diagnosis** | **Surgical procedure** | **Histological type** | **Pathologic T AJCC, 7th edition** | **Pathologic N AJCC, 7th edition** | **Grade** | **Anatomical region** | **Other features of interest** | **Molecular subtype** | **Neoadjuvant treatment** | **Reported recurrence or death in 12 months follow up** |
| 5931 | 75 | Male | Adenocarcinoma | Subtotal gastrectomy | Intestinal invasive adenocarcinoma | pT3 | pN0 | Moderate to poorly differentiated | Gastric | Background intestinal metaplasia | MSI | None | No |
| 6207 | 70 | Female | Adenocarcinoma | Distal partial gastrectomy | Intestinal adenocarcinoma | ypT4 | ypN0 | Moderately differentiated | Body and antrum invovled | Intestinal metaplasia with acute and chronic inflammation | MSI | Neoadjuvant with poor response, score 3 | No |

| **TableS2. The clinicopathologic parameters of the patients in phs001818.v2** | | | | | | | | | | | | |  |
| --- | --- | --- | --- | --- | --- | --- | --- | --- | --- | --- | --- | --- | --- |
| **Patient ID** | | | **Age** | **Sex** | **Histological diagnosis** | | **Smoking  (cigs/day)** | **Alcohol  (units/week)** | **Family  history of  GC** | | **H. pylori  positive*** | |  |
| P1 | | | 58 | male | Non Atrophic Gastritis | | 0 | 0 | N | | N | |  |
| P2 | | | 56 | Female | Non Atrophic Gastritis | | 0 | 0 | N | | N | |  |
| P8 | | | 67 | male | Non Atrophic Gastritis | | 0 | 1 | N | | N | |  |
| P9 | | | 62 | male | Early intestinal Gastric Cancer | | 1 | 0 | N | | N | |  |
| *P: H. pylori positive, N: H. pylori negative. | | |  |  |  | |  |  |  | |  | |  |
| **TableS3. Gene Ontology analysis of DEGs in gastric cancer** | | | | | | | | | | | | | |
| **GO analysis** | **Term ID** | **Description** | | | | **Gene Count** | | | | **GeneRatio** | | P.adjust | |
| BP | GO:0043062 | extracellular structure organization | | | | 27 | | | | 27/202 | | 0.000000303 | |
| BP | GO:0030198 | extracellular matrix organization | | | | 23 | | | | 23/202 | | 0.00000538 | |
| BP | GO:0006936 | muscle contraction | | | | 20 | | | | 20/202 | | 0.000353248 | |
| BP | GO:0006805 | xenobiotic metabolic process | | | | 11 | | | | 11/202 | | 0.000430648 | |
| BP | GO:0003012 | muscle system process | | | | 21 | | | | 21/202 | | 0.001766336 | |
| CC | GO:0062023 | collagen-containing extracellular matrix | | | | 29 | | | | 29/211 | | 9.32E-10 | |
| CC | GO:0031012 | extracellular matrix | | | | 31 | | | | 31/211 | | 9.32E-10 | |
| CC | GO:0098644 | complex of collagen trimers | | | | 7 | | | | 7/211 | | 0.00000103 | |
| CC | GO:0005583 | fibrillar collagen trimer | | | | 5 | | | | 5/211 | | 0.000027 | |
| CC | GO:0098643 | banded collagen fibril | | | | 5 | | | | 5/211 | | 0.000027 | |
| MF | GO:0005201 | extracellular matrix structural constituent | | | | 16 | | | | 16/201 | | 0.00000112 | |
| MF | GO:0030020 | extracellular matrix structural constituent conferring tensile strength | | | | 8 | | | | 8/201 | | 0.0000158 | |
| MF | GO:0005198 | structural molecule activity | | | | 27 | | | | 27/201 | | 0.0000309 | |
| MF | GO:0048407 | platelet-derived growth factor binding | | | | 4 | | | | 4/201 | | 0.002448497 | |
| MF | GO:0005539 | glycosaminoglycan binding | | | | 12 | | | | 12/201 | | 0.002448497 | |

| **TableS4. KEGG pathway analysis of DEGs in gastric cancer** | | | | |  |
| --- | --- | --- | --- | --- | --- |
| **Category** | **Term ID** | **Description** | **Gene Count** | **GeneRatio** | ***P***.adjust |
| KEGG pathway | hsa04974 | Protein digestion and absorption | 10 | 10/111 | 6.98E-05 |
|  | hsa05204 | Chemical carcinogenesis | 9 | 9/111 | 0.000149855 |
|  | hsa00982 | Drug metabolism - cytochrome P450 | 8 | 8/111 | 0.000347251 |
|  | hsa00980 | Metabolism of xenobiotics by cytochrome P450 | 8 | 8/111 | 0.000391572 |
|  | hsa00830 | Retinol metabolism | 7 | 7/111 | 0.001206052 |
|  | hsa04971 | Gastric acid secretion | 7 | 7/111 | 0.002091047 |
|  | hsa04657 | IL-17 signaling pathway | 7 | 7/111 | 0.006948443 |
|  | hsa00591 | Linoleic acid metabolism | 4 | 4/111 | 0.012868747 |
|  | hsa04512 | ECM-receptor interaction | 6 | 6/111 | 0.021810277 |
|  | hsa00140 | Steroid hormone biosynthesis | 5 | 5/111 | 0.022801899 |

| **Table S5. Association of hub genes expression with clinicopathological parameters of GC based on TCGA data** | | | | | | | | | | | | | | | | | |
| --- | --- | --- | --- | --- | --- | --- | --- | --- | --- | --- | --- | --- | --- | --- | --- | --- | --- |
| **Gene** | **Expression** | ***P*** | **TNM stage** | | ***P*** | **T** | | ***P*** | **N** | | ***P*** | **M** | | ***P*** | **Grade** | | ***P*** |
|  |  |  | **I-II** | **III-IV** |  | **T1-T2** | **T3-T4** |  | **Absence** | **Presence** |  | **Absence** | **Presence** |  | **G1-G2** | **G3-G4** |  |
| MMP9 | low | **<0.001** | 34 | 54 | 0.188 | 16 | 72 | 0.075 | 25 | 63 | 0.615 | 81 | 7 | 0.543 | 30 | 58 | 0.662 |
|  | high |  | 123 | 136 |  | 74 | 185 |  | 83 | 176 |  | 245 | 14 |  | 97 | 162 |  |
| COL1A1 | low | **<0.001** | 40 | 48 | 1.000 | 30 | 58 | 0.060 | 30 | 58 | 0.574 | 82 | 6 | 0.928 | 38 | 50 | 0.175 |
|  | high |  | 117 | 142 |  | 60 | 199 |  | 78 | 181 |  | 244 | 15 |  | 89 | 170 |  |
| COL1A2 | low | **<0.001** | 39 | 49 | 0.938 | 30 | 58 | 0.060 | 28 | 60 | 0.976 | 83 | 5 | 1.000 | 44 | 44 | **0.004** |
|  | high |  | 118 | 141 |  | 60 | 199 |  | 80 | 179 |  | 243 | 16 |  | 83 | 176 |  |
| TIMP1 | low | **<0.001** | 42 | 50 | 1.000 | 33 | 59 | **0.017** | 32 | 60 | 0.452 | 89 | 3 | 0.292 | 41 | 51 | 0.085 |
|  | high |  | 115 | 140 |  | 57 | 198 |  | 76 | 179 |  | 237 | 18 |  | 86 | 169 |  |
| SPP1 | low | **<0.001** | 37 | 50 | 0.643 | 22 | 65 | 0.985 | 25 | 62 | 0.673 | 85 | 2 | 0.151 | 29 | 58 | 0.547 |
|  | high |  | 120 | 140 |  | 68 | 192 |  | 83 | 177 |  | 241 | 19 |  | 98 | 162 |  |
| THBS2 | low | **<0.001** | 38 | 48 | 0.918 | 26 | 60 | 0.365 | 24 | 62 | 0.543 | 83 | 3 | 0.374 | 39 | 47 | 0.070 |
|  | high |  | 119 | 142 |  | 64 | 197 |  | 84 | 177 |  | 243 | 18 |  | 88 | 173 |  |
| VCAN | low | **<0.001** | 43 | 45 | 0.506 | 31 | 57 | **0.031** | 31 | 57 | 0.407 | 82 | 6 | 0.928 | 48 | 40 | **<0.001** |
|  | high |  | 114 | 145 |  | 59 | 200 |  | 77 | 182 |  | 244 | 15 |  | 79 | 180 |  |
| BGN | low | **<0.001** | 38 | 51 | 0.662 | 29 | 60 | 0.129 | 24 | 65 | 0.396 | 86 | 3 | 0.331 | 44 | 45 | **0.005** |
|  | high |  | 119 | 139 |  | 61 | 197 |  | 84 | 174 |  | 240 | 18 |  | 83 | 175 |  |
| SERPINE1 | low | **<0.001** | 40 | 44 | 0.707 | 25 | 59 | 0.438 | 26 | 58 | 1.000 | 80 | 4 | 0.759 | 38 | 46 | 0.079 |
|  | high |  | 117 | 146 |  | 65 | 198 |  | 82 | 181 |  | 246 | 17 |  | 89 | 174 |  |
| COL4A1 | low | **<0.001** | 41 | 47 | 0.865 | 29 | 59 | 0.110 | 26 | 62 | 0.813 | 85 | 3 | 0.345 | 36 | 52 | 0.399 |
|  | high |  | 116 | 143 |  | 61 | 198 |  | 82 | 177 |  | 241 | 18 |  | 91 | 168 |  |
| COL5A2 | low | **<0.001** | 46 | 44 | 0.240 | 32 | 58 | **0.023** | 30 | 60 | 0.694 | 86 | 4 | 0.627 | 41 | 49 | 0.055 |
|  | high |  | 111 | 146 |  | 58 | 199 |  | 78 | 179 |  | 240 | 17 |  | 86 | 171 |  |
| COL5A1 | low | **<0.001** | 41 | 47 | 0.865 | 30 | 58 | 0.060 | 28 | 60 | 0.976 | 82 | 6 | 0.928 | 42 | 46 | **0.017** |
|  | high |  | 116 | 143 |  | 60 | 199 |  | 80 | 179 |  | 244 | 15 |  | 85 | 174 |  |
| SPARC | low | **<0.001** | 40 | 48 | 1.000 | 31 | 57 | **0.031** | 25 | 63 | 0.615 | 85 | 3 | 0.345 | 39 | 49 | 0.107 |
|  | high |  | 117 | 142 |  | 59 | 200 |  | 83 | 176 |  | 241 | 18 |  | 88 | 171 |  |
| COL12A1 | low | **<0.001** | 39 | 49 | 0.938 | 25 | 63 | 0.637 | 26 | 62 | 0.813 | 83 | 5 | 1.000 | 32 | 56 | 1.000 |
|  | high |  | 118 | 141 |  | 65 | 194 |  | 82 | 177 |  | 243 | 16 |  | 95 | 164 |  |
| COL11A1 | low | **<0.001** | 44 | 43 | 0.303 | 32 | 55 | **0.012** | 28 | 59 | 0.910 | 82 | 5 | 1.000 | 37 | 50 | 0.231 |
|  | high |  | 113 | 147 |  | 58 | 202 |  | 80 | 180 |  | 244 | 16 |  | 90 | 170 |  |
| COMP | low | **<0.001** | 40 | 50 | 0.957 | 26 | 64 | 0.547 | 27 | 63 | 0.892 | 86 | 4 | 0.627 | 42 | 48 | **0.030** |
|  | high |  | 117 | 140 |  | 64 | 193 |  | 81 | 176 |  | 240 | 17 |  | 85 | 172 |  |
| ADAMTS2 | low | **<0.001** | 41 | 47 | 0.865 | 25 | 63 | 0.637 | 30 | 58 | 0.574 | 81 | 7 | 0.543 | 48 | 40 | **<0.001** |
|  | high |  | 116 | 143 |  | 65 | 194 |  | 78 | 181 |  | 245 | 14 |  | 79 | 180 |  |

| **Table S6.The correlation between the drugs and hub genes** | | | |
| --- | --- | --- | --- |
| **Gene** | **Drug** | **cor** | **pvalue** |
| SPARC | By-Product of CUDC-305 | -0.492636024 | 6.38E-05 |
| COMP | Thiotepa | 0.42121508 | 0.00080379 |
| COL5A2 | Hydrastinine HCl | 0.420013338 | 0.000834982 |
| COMP | Idarubicin | 0.419947753 | 0.000836715 |
| COMP | Triethylenemelamine | 0.412153745 | 0.0010674 |
| SPARC | Zoledronate | 0.396785304 | 0.001696615 |
| SPARC | Bleomycin | 0.39076565 | 0.00202236 |
| COMP | Valrubicin | 0.384481327 | 0.002420988 |
| COMP | Mitomycin | 0.380812041 | 0.002684837 |
| COL5A2 | okadaic acid | 0.378072438 | 0.002898211 |
| BGN | Zoledronate | 0.37580777 | 0.003085836 |
| COMP | Bendamustine | 0.374089257 | 0.003235331 |
| SPARC | 8-Chloro-adenosine | -0.364939085 | 0.004144665 |
| BGN | LY-294002 | 0.358253302 | 0.004945111 |
| BGN | Cobimetinib (isomer 1) | -0.353520642 | 0.005591153 |
| COL5A2 | LY-294002 | 0.344464508 | 0.007036636 |
| COL5A2 | Bleomycin | 0.343520022 | 0.007204727 |
| COMP | XK-469 | 0.343304606 | 0.007243552 |
| COMP | Pipobroman | 0.342806815 | 0.007333972 |
| BGN | Trametinib | -0.341503356 | 0.007575421 |
| COMP | Hydroxyurea | 0.338129506 | 0.008232878 |
| COL5A2 | BEN | 0.336995811 | 0.008464691 |
| COMP | Teniposide | 0.336478257 | 0.008572394 |
| COMP | Irinotecan | 0.33601471 | 0.008669869 |
| SPARC | LDK-378 | -0.333824634 | 0.009143556 |
| BGN | By-Product of CUDC-305 | -0.327568734 | 0.010622597 |
| BGN | LOR-253 | -0.326234813 | 0.01096348 |
| COL5A2 | Abiraterone | 0.325424378 | 0.011175175 |
| COL5A2 | Palbociclib | -0.322743306 | 0.011900891 |
| SPARC | Lenvatinib | 0.321326198 | 0.012300657 |
| COL5A2 | Carboplatin | 0.317876618 | 0.013322515 |
| COMP | Chlorambucil | 0.317447563 | 0.013454582 |
| COMP | Etoposide | 0.31712278 | 0.013555301 |
| BGN | bisacodyl, active ingredient of viraplex | 0.314899367 | 0.014262386 |
| COL5A2 | Allopurinol | -0.31375641 | 0.014638037 |
| SPARC | Simvastatin | 0.312330737 | 0.015118486 |
| SPARC | Palbociclib | -0.312323156 | 0.015121076 |
| SPARC | Staurosporine | 0.311757584 | 0.015315413 |
| COMP | Cytarabine | 0.309652706 | 0.016057601 |
| SPARC | Dexrazoxane | -0.308080257 | 0.016631951 |
| COL5A2 | Cobimetinib (isomer 1) | -0.305499277 | 0.017612751 |
| BGN | Actinomycin D | -0.305495066 | 0.017614391 |
| COMP | Uracil mustard | 0.303844204 | 0.018267334 |
| BGN | Alvespimycin | -0.303104957 | 0.018566341 |
| SPARC | Allopurinol | -0.302239664 | 0.018921619 |
| COMP | LMP-400 | 0.30216052 | 0.018954402 |
| BGN | Nitrogen mustard | -0.301219542 | 0.019347887 |
| COMP | RH1 | 0.300474661 | 0.019664281 |
| BGN | Selumetinib | -0.298338164 | 0.020596287 |
| COMP | Oxaliplatin | 0.297660585 | 0.020899592 |
| COL5A2 | By-Product of CUDC-305 | -0.296336737 | 0.021503134 |
| SPARC | Fulvestrant | -0.2963074 | 0.021516675 |
| COMP | Nitrogen mustard | 0.294341908 | 0.022440452 |
| SPARC | Cabozantinib | 0.292840158 | 0.0231687 |
| SPARC | Gemcitabine | 0.291835352 | 0.023667041 |
| COMP | Cisplatin | 0.290584662 | 0.024299954 |
| BGN | Palbociclib | -0.288251559 | 0.025518834 |
| COL5A2 | Idelalisib | 0.288041989 | 0.025630797 |
| COMP | Triapine | 0.287363946 | 0.025995881 |
| BGN | Amonafide | -0.286637413 | 0.026391923 |
| COL5A2 | Rapamycin | 0.286151498 | 0.026659624 |
| COMP | Mitoxantrone | 0.281686723 | 0.029227988 |
| COMP | Batracylin | 0.277496289 | 0.031824051 |
| SPARC | Tamoxifen | -0.277030864 | 0.032123916 |
| COMP | Topotecan | 0.276991273 | 0.032149532 |
| COMP | LMP776 | 0.27642194 | 0.032519792 |
| COMP | (+)-JQ1 | -0.274771969 | 0.033612977 |
| BGN | ABT-199 | -0.274006793 | 0.034130235 |
| COL5A2 | Salinomycin | 0.273948399 | 0.03416998 |
| SPARC | Vemurafenib | 0.270991324 | 0.036233621 |
| SPARC | Vinorelbine | -0.270331796 | 0.03670772 |
| SPARC | Idelalisib | 0.270244514 | 0.036770846 |
| SPARC | Docetaxel | -0.269988403 | 0.036956597 |
| SPARC | Alvespimycin | -0.268595189 | 0.037980737 |
| COMP | 5-fluoro deoxy uridine 10mer | 0.266339856 | 0.039688371 |
| COL5A2 | Lificguat | -0.266137505 | 0.039844635 |
| COMP | By-Product of CUDC-305 | 0.264161535 | 0.041397447 |
| COL5A2 | Irofulven | 0.263965958 | 0.041553819 |
| COL5A2 | Everolimus | 0.263510937 | 0.041919511 |
| COL5A2 | Cisplatin | 0.261839677 | 0.043285503 |
| SPARC | Eribulin mesilate | -0.260667112 | 0.044265587 |
| COMP | Epirubicin | 0.260304862 | 0.044572034 |
| COL5A2 | Temsirolimus | 0.259569065 | 0.045199851 |
| COL5A2 | Ethinyl estradiol | 0.25817845 | 0.046406212 |
| COMP | Raltitrexed | 0.258109038 | 0.046467112 |
| SPARC | Wortmannin | 0.257683491 | 0.046841908 |
| SPARC | Belinostat | -0.254331778 | 0.049881283 |

**TableS7.Changes in the number of cells before and after quality control in each sample**

|  | Cancer1 | Cancer2 | Cancer3 | Normal1 | Normal2 | Normal3 | Total |
| --- | --- | --- | --- | --- | --- | --- | --- |
| Before QC | 7108 | 2515 | 4110 | 2774 | 1998 | 1865 | 20370 |
| After QC | 3885 | 2104 | 2578 | 2212 | 1702 | 1358 | 13839 |
